# Paraoxonase-1 (PON-1) Arylesterase Activity Levels in Patients with Coronary Artery Disease: A Meta-Analysis

**DOI:** 10.1155/2022/4264314

**Published:** 2022-03-10

**Authors:** Marco Zuin, Alessandro Trentini, Judit Marsillach, Andrea D'Amuri, Cristina Bosi, Loris Roncon, Angelina Passaro, Giovanni Zuliani, Mike Mackness, Carlo Cervellati

**Affiliations:** ^1^Department of Translational Medicine and for Romagna, University of Ferrara, Via Luigi Borsari 46, 44121 Ferrara, Italy; ^2^Department of Environmental and Prevention Sciences, University of Ferrara, Via Luigi Borsari 46, 44121, Italy; ^3^Medicine (Division of Medical Genetics), University of Washington, Health Sciences Building, Seattle, WA 98195-7720, USA; ^4^Medical Department, University Hospital of Ferrara Arcispedale Sant'Anna, Via Aldo Moro 8, 44124 Ferrara, Italy; ^5^Department of Cardiology, Santa Maria della Misericordia Hospital, Viale Tre Martiri 140, 45100 Rovigo, Italy; ^6^Research and Innovation Section, University Hospital of Ferrara Arcispedale Sant'Anna, Via Aldo Moro 8, 44124 Ferrara, Italy; ^7^Avenida Principe De' Espanya, Miami Platja 43892, Tarragona, Spain

## Abstract

**Aim:**

To review and compare the PON-1 arylesterase activity between coronary artery disease (CAD) and non-CAD patients.

**Methods:**

Data were obtained by searching MEDLINE and Scopus for all investigations published between January 1, 2000 and March 1, 2021 comparing PON-1 arylesterase activity between CAD and controls.

**Results:**

Twenty studies, based on 5417 patients, met the inclusion criteria and were included in the analysis. A random effect model revealed that PON-1 arylesterase activity was significantly lower in the CAD group compared to controls (SMD = –0.587, 95%CI = −0.776 to -0.339, *p* < 0.0001, *I*^2^ = 92.3%). In CAD patients, the PON-1 arylesterase activity was significantly higher among CAD patients without diabetes mellitus (DM) compared to those with diabetes (SMD: 0.235, 95% CI: 0.014 to 0.456, *p* = 0.03, *I*^2^ = 0%).

**Conclusions:**

PON-1 activity is significantly lower in CAD patients, and those without DM presented a significantly higher PON-1 arylesterase activity.

## 1. Introduction

Coronary artery disease (CAD) remains the leading cause of morbidity and mortality worldwide [1]. Over the last decades, several novel biochemical markers of oxidative stress and related genetic polymorphisms have been identified in patients with CAD [2]. Indeed, free radicals contribute to endothelial dysfunction (ED) and to the oxidation of low-density lipoproteins (LDL), which are both critical pathogenic events of atherosclerosis [3]. Furthermore, it has been widely reported that elevated concentration of several oxidative stress markers is linked with a higher incidence of cardiovascular events [4]. Paraoxonase-1 (PON-1) is an enzyme that has many enzymatic activities, such as lactonase, thiolactonase, arylesterase, and aryldialkylphosphatase (commonly known as paraoxonase). The most important physiological role of PON-1 is the ability to hydrolyse oxidized LDL (ox-LDL) and thereby delay the onset of atherosclerosis [5]. PON-1 is classified as an accessory protein of high-density lipoprotein (HDL) and modulates the capacity of HDL to protect against the atherosclerosis process through antioxidant and anti-inflammatory activities [5]. Previous meta-analyses of the relationship between PON-1 and CAD have concentrated on PON-1 SNP's or PON-1 paraoxonase activity [6–8]. However, some studies have demonstrated that the arylesterase activity of PON-1 was decreased in CAD patients when compared to non-CAD subjects, thus confirming the earlier preclinical evidence [9]. However, to the best of our knowledge, these data have never been analyzed comprehensively. Therefore, the aim of the present study is to perform a systematic review and meta-analysis comparing the serum/plasma PON-1 activity, measured as arylesterase activity between CAD and non-CAD patients.

## 2. Materials and Methods

### 2.1. Study Design and Eligibility Criteria

This study followed the Preferred Reporting Items for Systematic Reviews and Meta-analyses (PRISMA) reporting guideline (Online Resource 1) [10]. Data were obtained searching MEDLINE and Scopus for all investigations published between January 1, 2000 and March 1, 2021 comparing the PON-1 arylesterase activity between CAD and controls.

### 2.2. Outcomes

The primary outcome was the comparison of PON-1 arylesterase plasma/serum levels between CAD and non-CAD patients, whereas the secondary objective of the analysis was to compare the arylesterase activity in CAD patients with or without diabetes mellitus (DM). Metaregression using as moderator variables age, body mass index (BMI), and the latitude of the study site was also carried out.

### 2.3. Data Extraction and Quality Assessment

The selection of studies to be included in our analysis was independently conducted by 2 authors (M.Z. and C.C.) in a blinded fashion. Any discrepancies in study selection were resolved by consulting a third author (A.T.). The following MeSH terms were used for the search: “Paraoxonase -1” AND “Coronary artery disease” OR “Coronary Heart disease.” Additionally, all references cited were reviewed to identify further studies that were not included in the abovementioned electronic databases. Studies were considered eligible if they provided data regarding PON-1 activity in both CAD and controls. Conversely, they were excluded from the analysis if (1) they did not provide a comparison between PON-1 arylesterase activity between CAD patients and controls; (2) they were case reports, review articles, abstracts, editorials/letters, randomized controlled trials, and case series with less than 15 participants from the general population or (3) they were not in the English language. Data extraction was independently conducted by 2 authors (M.Z. and C.C.). Discrepancies between reviewers were resolved by consensus. For all studies reviewed, we extracted the number of patients enrolled, the mean age, male gender, mean and standard deviation (SD) of PON-1 activity levels, and prevalence of traditional cardiovascular risk factors among CAD and non-CAD subjects. Newcastle-Ottawa scale (NOS) was used to evaluate the methodology quality of eligible studies [11].

### 2.4. Data Synthesis and Analysis

Continuous variables were expressed as mean ± (SD) or as median with corresponding interquartile range while categorical variables were expressed as counts and percentages. The difference of PON-1 arylesterase activity level between CAD and controls was expressed as standardized mean difference (SMD) with the corresponding 95% confidence interval (CI) using a random-effect model (DerSimonian-Laird). A *I*^2^ = 0 was considered to indicate no heterogeneity while values of *I*^2^ as <25%, 25–75%, and above 75% indicate low, moderate, and high degrees of heterogeneity, respectively [12]. When significant publication bias was found, we used the trim-and-fill method to adjust our results. To evaluate publication bias both funnel plot and Egger's test were computed. To further appraise the impact of potential baseline confounders, a metaregression analysis using age, body mass index (BMI), gender, HDL-C, and the latitude of the study site as moderator variables was performed. A further subanalysis was also performed to assess any difference in PON-1 arylesterase activity among Asian and European populations from the reviewed studies. The meta-analysis was conducted using Comprehensive Meta-Analysis software, version 3 (Biostat, USA).

## 3. Results

### 3.1. Search Results and Included Studies

**Figure 1:**
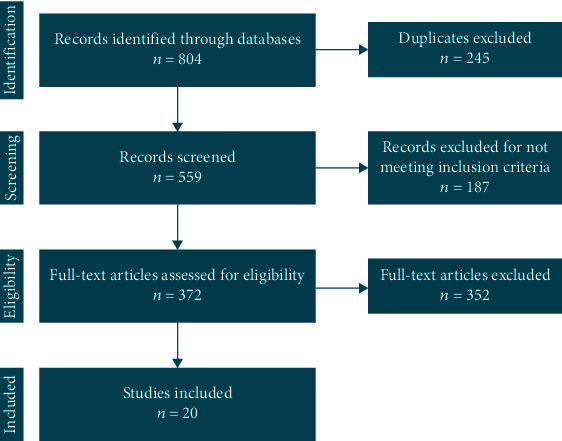
PRISMA flow chart.

A total of 804 articles were obtained with our search strategy. After excluding duplicates and preliminary screening, 372 full-text articles were assessed for eligibility, and 352 studies were excluded for not meeting the inclusion criteria, leaving 20 investigations fulfilling the inclusion criteria (Figure 1) [9, 13–30].

### 3.2. Characteristics of the Population and Quality Assessment

Overall, 5 417 patients (3 364 with CAD and 2 053 without CAD) were included in the analysis. The general characteristics of the studies included were the relative demographic, biometrical, and lipid profiles shown in Table 1. Quality assessment showed that all studies were of moderate-high quality according to the NOS scale (Online Resource 2).

### 3.3. PON-1 Arylesterase Activity in Patients with Coronary Artery Disease

**Figure 2:**
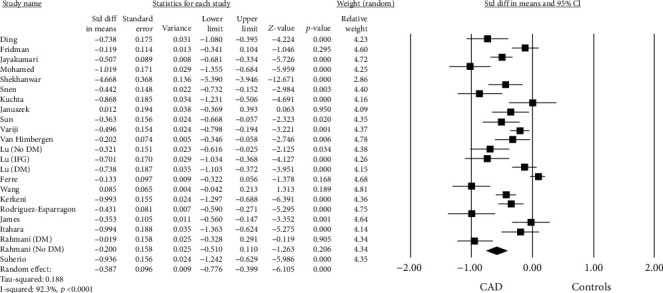
Forest plot investigating the PON-1 arylesterase activity in coronary artery disease (CAD) patients and controls.

A random effect model revealed that PON-1 arylesterase activity was significantly lower in the CAD group compared to controls (SMD = –0.587, 95%CI = −0.776 to − 0.339, *p* < 0.0001, *I*^2^ = 92.3%) (Figure 2). Egger's tests (*t* = 4.286, *p* = 0.003) showed evidence of potential publication bias. Therefore, a trim-and-fill analysis was performed to explore whether the publication bias influenced the stability of the results in this meta-analysis (two studies trimmed). The updated result showed a SMD = −0.881 (95% CI -1.139 to -0.624, *p* < 0.0001). The relative funnel plot is showed in Supplementary file 3 (Panel A).

**Figure 3:**
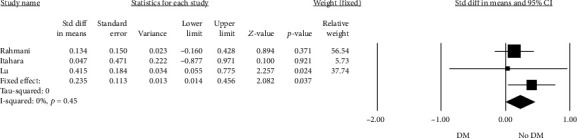
Forest plot of the PON-1 arylesterase activity in subjects with diabetes mellitus (DM) or nondiseased (no DM).

A subgroup analysis was performed in order to estimate the possible existence of a difference in PON-1 arylesterase activity in CAD patients having or not having DM; PON-1 arylesterase activity was significantly higher among CAD patients without DM compared to those with diabetes (SMD: 0.235, 95% CI: 0.014 to 0.456, *p* = 0.03, *I*^2^ = 0%) (Figure 3). In this case, Egger's test revealed no evidence of publication bias (*t* = 0.115, *p* = 0.927). The relative funnel plot is presented in Online Resource 3, panel B.

### 3.4. PON-1 Arylesterase Activity in Patients with Coronary Artery Disease by Geographical Area

Using a random effect model, a further subanalysis revealed that PON-1 arylesterase activity remained significant lower in patients with CAD both in Asian (SMD: -0.558, 95% CI: -0.917 to -0.200, *p* = 0.002, *I*^2^: 91%) and European (SMD: -0.298, 95% CI: -0.497 to -0.015, *p* = 0.003, *I*^2^ = 90%) populations (forest plot shown in Online Resource 4). Although the diabetes status of patients enrolled was not systematically evaluated, from those studies that did record diabetes status, the prevalence of diabetes was higher amongst CHD patients. Notably, Asian patients with CHD were more frequently diabetic than their European counterparts (47.0% vs. 32.3%).

### 3.5. Metaregression

In metaregression analysis, no correlation was found between either SMD and age (*p* = 0.13), BMI (*p* = 0.09), gender (*p* = 0.24), and latitude of the study site (*p* = 0.80), while an inverse association was found using HDL-C levels (*p* = 0.004) and statin treatment (*p* = 0.003) as moderators (Table 2).

## 4. Discussion

The present meta-analysis revealed a significant decrease in PON-1 arylesterase activity in CAD patients compared to controls. For the first time, our results provide a comprehensive and updated evaluation of PON-1 arylesterase activity in CAD patients, reviewing all the available studies published over the last twenty years. Indeed, several investigations have been previously focused on the relationship between PON-1 single nucleotide polymorphisms (SNPs) and cardiovascular disease (CVD) [31–33], including CAD [7]. However, the results reported in these studies were highly variable, with some of them revealing either a significant or a nonsignificant association between genetic variants and disease occurrence. The most common SNPs of this HDL-associated protein (Q192R and L155M), mostly influence the so-called paraoxonase activity, are responsible for hydrolysis of certain organophosphate compounds. Indeed, the high interindividual variability of this activity is essentially due to the effect of these genetic variants. On the other hand, arylesterase, although not being the physiological activity, has been found to more properly reflect the antiatherosclerotic properties of PON-1 [34]. This activity is by far the most frequently assessed in epidemiological-clinical studies also because of its lower interindividual variability.

Despite intense research on the topic, the biology and biochemistry of PON-1 are still poorly understood. Indeed, the physiological substrate has not been definitively ascertained; although, the most recent in vitro evidence points to endogenous lipophilic lactones such as those resulting from fatty acid oxidation (e.g., 5,6-dihydroxy-trienoic acid and 1,5-lactone). Unfortunately, at the current state of the art, a universally accepted assay to measure the (putative) native lactonase activity has not yet been validated, making a meta-analysis on this PON-1 activity unfeasible. The cause/effect relationship between PON-1 and CAD (along with other diseases) is also unclear. The enzyme structure and activity are highly susceptible of oxidative and glycation modification. Thus, the decrease in PON-1 observed in diabetic CAD patients compared to nondiabetic could be both a downstream and upstream event linked to either glycation or oxidative stresses that characterize the metabolic disease. The interest in the biological role of PON-1 was sparked by converging in vivo and in vitro evidence suggesting that this protein is able to protect lipid moiety of cell membranes and LDL from oxidation [35–37]. Mackness and colleagues were the first to demonstrate that PON-1 could prevent the accumulation of lipid peroxides in low-density lipoproteins (LDL) [38]. Afterwards, studies on transgenic mice confirmed and extended these findings. PON-1 knockout mice showed increased serum and arterial macrophage oxidative stress and an increase in atherosclerotic lesions compared to controls; moreover, the addition of PON-1 to macrophages significantly improved their redox imbalance [37].

These compelling preclinical findings provide the rationale of the cross-sectional studies included in our analysis, as well as of longitudinal studies investigating the link of arylesterase and/or paraoxonase activity with CVD risk. To the best of our knowledge, the largest longitudinal study (PREVEND study, mean follow − up = 9.3 years) in this field found an approximately log-linear inverse association between the two variables, which was partly dependent on HDL-C levels [39]. This result is not surprising, since PON-1 is mostly bound to HDL particles, and PON-1 activity is strongly correlated to the level of cholesterol carried by the lipoprotein. As also confirmed by the outcome of the significant inverse association between HDL-C and PON-1 revealed by the meta-regression analysis, the level of the former marker should be always considered as potential confounding factor in all clinical studies dealing with PON-1. The authors of PREVEND study also performed a meta-analysis, including two other studies. This further analysis showed that PON-1 arylesterase did not provide significant improvement in CVD risk assessment beyond conventional CVD risk factors [6]. However, it should be noted that the definition of CV risk encompasses several underlying acute and/or chronic conditions, which are influenced by other modifiable and nonmodifiable determinants. In this regard, our analysis specifically focused on CAD, which represents the primary cause of mortality worldwide.

Our meta-regression suggests that, besides HDL-C (or other surrogate markers of HDL concentration, such as Apo A1), statin use should also be considered in the multivariate analysis on the link between PON-1 and CAD occurrence. This outcome agrees with a recent meta-analysis showing that statin therapy may have a positive effect in improving both PON-1 paraoxonase and arylesterase activities, either in single-arm studies or controlled trials [39]. To explain this finding, the authors hypothesized that these cholesterol lowering drugs may enhance PON-1 protein synthesis and secretion or interaction with HDL [40]. Conversely, no association with BMI, age, or gender was observed. These findings might reflect the characteristics of the subjects enrolled in the reviewed studies. Indeed, most of the patients were mid-age individuals, and BMI was not systematically reported, probably underestimating the potential effects of such variables. Indeed, to really assess the role of age in the association between PON-1 and CAD, it would be useful to include in the cohorts also younger subjects, to really assess the trend of the interaction over the decades, starting when CAD patients typically become symptomatic [41].

Serum evaluation of PON-1 may represent a useful adjunctive tool in different clinical scenarios. First, it could be used to early identify young patients with subclinical atherosclerosis, thus integrating the traditional cardiovascular risk assessment based on the lipid profile evaluation, blood pressure measurement, and intima media-thickness assessment. Second, it might be evaluated in secondary CV prevention, demonstrating the possible antioxidant effects of statins against lipid peroxidation via lipid-lowering-dependent and -independent mechanisms [42].

### 4.1. Limitations

Our study has several limitations related to the observational nature of the studies reviewed with all the inherited biases. In fact, the high heterogeneity observed, which probably depends on the participants' inclusion criteria as well as on the study designs, may have resulted in conclusions that are not firm. Furthermore, the presence of publication bias, despite the application of the trim-and-fill method, may have also confounded the results. In addition, most of the studies considered in the analysis lacked information regarding the use of statins and other drugs, which have been suggested to influence PON-1 expression and activity. This is an important point, since it has been shown that different types of statins may exert different effects on PON-1 expression/activity [45]. Finally, the number of studies reporting data about CAD patients with concomitant DM is limited, and this may have affected the reliability of the findings on this subtopic.

## 5. Conclusion

Despite the acknowledged limitations, our findings clearly suggest that patients suffering from CAD have a decreased PON-1 arylesterase. Further research efforts are required to ascertain whether change of this activity may precede and thus predict disease occurrence.

## Figures and Tables

**Table 1:** General characteristics of the population enrolled.

Author	Study type	Country	CAD, *N* (% male)	Non CAD, *N* (% male)	Mean age (years)		BMI (kg/m^2^)		TC mmol/L [mg/dl]		C-HDL mmol/L [mg/dl]		TG mmol/L [mg/dl]	
					CAD	Non-CAD	CAD	Non-CAD	CAD	Non-CAD	CAD	Non-CAD	CAD	Non-CAD
Sun et al. [9]	Case-control	China	130 (50)	47 (53)	67.0 ± 11.0	65.6 ± 11.0	21.3 ± 2.1	20.4 ± 14	4.54 ± 1.28	4.55 ± 0.9	1.15 ± 0.33	1.28 ± 0.31	1.45 ± 0.81	1.32 ± 0.68

Fridman et al. [13]	Case-control	Argentina	286 (37)	245 (34)	63.4 ± 1.5	60.0 ± 1.3	NR		5.18 ± 0.15	5.10 ± 0.08	1.58 ± 0.03	1.65 ± 0.03^∗^	2.00 ± 0.16	1.62 ± 0.09^∗^

Jayakumari et al. [24]	Case-control	India	284 (100)	245 (100)	51.0 ± 6.9	44.0 ± 10.2	NR		[206 ± 42]	[220 ± 44]	[33 ± 7.7]	[42 ± 9.5]	[164 ± 72]	[141 ± 68]^∗^

Mohamed et al. ^−^[25]	Retrospective cohort	Egypt	150 (79)	50 (52)	55.5 ± 8.1	50.7 ± 9.5	NR		[240.5 ± 27.4]	[185.9 ± 8.4]^∗^	[42.1 ± 4.2]	[54.7 ± 3.2]^∗^	[175.7 ± 13.9]	[132.0 ± 14.4]^∗^

Shekhanawa et al. [26]	Cross-sectional	India	60 (67)	50 (58)	NR	NR	NR	NR	[222.6 ± 49.5]	[154 ± 32.7]	NR	NR	[206.157.6]	[123.0 ± 32.6]

Shen et al. [27]	Cross-sectional	China	144 (75)	69 (54)	64.8 ± 10.3	65.3 ± 9.1	25.5 ± 3.4	24.9 ± 3.1	4.3 ± 1.2	4.4 ± 1.0	1.01 ± 0.24	1.13 ± 0.22	1.82 ± 1.09	1.75 ± 0.96

Kuchta et al. [28]	Cross-sectional	Poland	105 (39)	45 (44)	65 ± 10	63 ± 10	28 ± 5	27 ± 4	[168 ± 41]	[196 ± 40]	[44 ± 11]	[52 ± 13]	107 (SD NR)	102 (SD NR)

Januszek et al. [29]	Case-control	Poland	53 (100)	53 (100)	51.3 ± 7.8	42.03 ± 4.82^∗∗^	29.4 ± 4.9	29.2 ± 3.8	6.02 ± 0.81	5.81 ± 0.95^∗^	1.29 ± 0.34	1.32 ± 0.41	2.11 ± 1.05	2.01 ± 1.42

Sun et al. [9]	Case-control	China	123 (63)	63 (44)	69.7 ± 11.3	62.7 ± 10.7^∗^	NR	NR	4.08 ± 1.07	4.50 ± 0.99^∗^	1.06 ± 0.32	1.14 ± 0.27^∗^	1.56 ± 0.38	1.69 ± 1.27

Variji et al. [30]	Case-control	Iran	126 (66)	203 (52)	63.4 ± 1.5	60.0 ± 1.3	NR	NR	5.18 ± 0.15	5.10 ± 0.08	1.52 ± 0.03	1.65 ± 0.03^∗^	2.00 ± 0.16	1.62 ± 0.09^∗^

van Himbergen et al. [14]	Case-cohort	Netherlands	211 (NR)	1527 (NR)	61 ± 6	57 ± 5^∗∗^	26.8 ± 3.9	25.8 ± 3.1^∗∗^	NR	NR	1.4 ± 0.3	1.6 ± 0.4^∗∗^	NR	NR

Lu et al. [15]	Case-control													

No DM cohort	-	China	88 (66)	90 (57)	55.6 ± 13.8	57.3 ± 8.6	NR	NR	4.85 ± 1.22	4.42 ± 1.21^∗^	1.19 ± 0.29	1.41 ± 0.38^∗^	1.90 ± 0.92	1.78 ± 1.01

IFG cohort	-	China	62 (100)	90 (57)	57.7 ± 11.4	57.3 ± 8.6	NR	NR	5.26 ± 1.25	4.42 ± 1.21^∗^	1.15 ± 0.35	1.41 ± 0.38^∗^	2.07 ± 0.99	1.78 ± 1.01

DM cohort	-	China	46 (57)	90 (57)	60.9 ± 11.5	57.3 ± 8.6	NR	NR	5.76 ± 2.11	4.42 ± 1.21^∗^	1.10 ± 0.1	1.41 ± 0.38^∗^	2.26 ± 1.23	1.78 ± 1.01

Ferrè et al. [16]	Case-control	Spain	215 (100)	215 (100)	60.6 ± 11.8	62.1 ± 16.4	27.1 ± 4.2	26.2 ± 3.5	5.74 ± 1.10^∗^	5.27 ± 1.2^∗^	1.11 ± 0.35	1.23 ± 0.42^∗^	1.97 ± 1.12	1.52 ± 0.91^∗^

Wang et al. [17]	Case-control	China	474 (100)	475 (100)	54.1 ± 8.9	53.8 ± 10.2	26.6 ± 2.9	24.2 ± 3.1	5.16 ± 1007	5.06 ± 0.94	1.06 ± 0.23	1.24 ± 0.30^∗^	1.73 ± 1.13	1.46 ± 1.0

Kerkeni et al. [18]	Case-control	Spain	100 (74)	120 (73)	59 ± 10	54 ± 10	28 ± 5	27.6 ± 5	4.94 ± 1.11	4.20 ± 1.03^∗∗^	0.73 ± 0.13	0.90 ± 0.24^∗∗^	1.49 ± 1.18	1.19 ± 0.68

Rodríguez-Esparragón et al. [19]	Case-control	Spain	304 (78)	315 (74)	56 ± 10	54.5 ± 16	27.2 ± 3.7	27.3 ± 3.8	5.2 ± 1.1	6.1 ± 1.0^∗∗^	0.92 ± 0.24	1.3 ± 0.32^∗∗^	1.24 ± 0.67	1.52 ± 0.7^∗^

James et al. [20]	Cross-sectional	Swiss	137 (76)	273 (64)	62.8 ± 9.6	59.0 ± 0.5^∗∗^	27.5 ± 4.1	27.9 ± 4.6	6.0 ± 1.1	5.9 ± 1.1	1.18 ± 0.6	1.3 ± 0.48	1.85 ± 0.9	1.7 ± 1.1
Itahara et al. [21]	Case-control	Japan	96 (59)	136 (52)	65 ± 11	63 ± 6	21.0 ± 31	22.7 ± 2.7^∗∗^	[146 ± 36]	[208±32]^∗∗^	[^∗∗^]	[60±14]^∗∗^	[106 ± 13]	[116 ± 64]

Rahmani et al. [22]	Cross-sectional	Iran											
DM cohort	**—**		89 (57)	73 (52)	56 ± 7.5	54.6 ± 7.7	27.3 ± 3.5	27.1 ± 4.3	[213 ± 38]	[196 ± 45]^∗^	[48 ± 13]	[51 ± 13]	[209 ± 187]	[150 ± 163]
No DM cohort	**—**		89 (57)	73 (52)	56.7 ± 7.0	54.6 ± 7.7	25.9 ± 4.0	27.1 ± 4.3^∗^	[205 ± 44]	[196 ± 45]	[48 ± 15]	[52 ± 13]	[173 ± 137]	[150 ± 163]

Suehiro et al. [23]	Case-control	Japan	81 (62)	103 (52)	64 ± 11	63 ± 11	21.2 ± 3.0	22.5 ± 2.0^∗^	[149 ± 38]	[206 ± 31]^∗^	[40 ± 13]	[58±13]^∗∗^	[106 ± +60]	[115±67]^∗∗^

CAD: coronary artery disease; BMI: body mass index; TC: total cholesterol; HDL: high-density lipoproteins; TG: triglycerides; DM: diabetes mellitus; IFG: impaired fasting glucose. The lipidic profile is presented with the measure's unit used in the original manuscript. ∗*p* < 0.05 vs controls; ∗∗*p* < 0.0001 vs. controls.

**Table 2:** Metaregression analyses.

Item	*N*° of interaction	*β*	SE	95% CI	*z* value	*p*
Age	22	-0.020	0.013	-0.047 to 0.006	-1.48	0.137
BMI	14	0.064	0.380	-0.010 to 0.139	1.68	0.092
Latitude	14	-0.003	0.012	-0.027 to 0.021	-0.25	0.805
Gender (females)	22	-0.0101	0.008	-0.271 to 0.007	-1.16	0.247
HDL-C	21	-1.913	0.670	-3.226 to -0.599	-2.85	0.004
Statin treatment	8	-1.958	0.702	-3.465 to -0.574	-2.91	0.003

*β*: standardized regression coefficient; SE: standard error; 95% CI: 95% confidence interval.

## Data Availability

Data sharing is not applicable to this article as no new data were created.

## Supplementary Materials

Supplementary Materials Supplementary file 1: PRISMA statement checklist. Supplementary File 2: quality of the included studies assessed using the Newcastle-Ottawa quality assessment scale (NOS). Supplementary File 3: funnel plots for PON-1 arylesterase activity in patients with coronary artery disease (CAD) in the presence or absence of diabetes. (a) Funnel plot for subjects with/withouth CAD. (b) Funnel plot for subjects with CAD but with/without diabetes. Supplementary file 4: forest plot analysis for CAD patients divided by geographical area. (a) Asian subjects. (b) European subjects.
